# High Sensitive and Non-invasive ctDNAs Sequencing Facilitate Clinical Diagnosis And Clinical Guidance of Non-small Cell Lung Cancer Patient: A Time Course Study

**DOI:** 10.3389/fonc.2018.00491

**Published:** 2018-10-30

**Authors:** Yongqiang Li, Jiajia Lv, Shaogui Wan, Junfang Xin, Tiantian Xie, Tao Li, Wan Zhu, Guosen Zhang, Yunlong Wang, Yitai Tang, Ao Li, Xiangqian Guo

**Affiliations:** ^1^Joint National Laboratory for Antibody Drug Engineering, Cell Signal Transduction Laboratory, Department of Preventive Medicine, School of Basic Medical Sciences, Institute of Biomedical Informatics, Henan University, Kaifeng, China; ^2^Laboratory of Cancer Biomarkers and Liquid Biopsy, Henan University, Kaifeng, China; ^3^Department of Anesthesia, Stanford University, Stanford, CA, United States; ^4^Henan Bioengineering Research Center, Zhengdong New District, Zhengzhou, China; ^5^Department of Pathology, Stanford University, Stanford, CA, United States; ^6^School of Information Science and Technology, Centers for Biomedical Engineering, University of Science and Technology of China, Hefei, China

**Keywords:** lung cancer, ctDNAs, NGS, non-invasive, ddPCR

## Abstract

Lung cancer is one of leading causes of cancer death all over the world. Non-small cell lung cancer (NSCLC) is the most predominant subtype of lung cancer. Molecular targeting therapy has been shown great success in the treatment of advanced NSCLC. Thus, an easy, sensitive, and specific way of recognizing therapeutic gene targets would help to select effective treatments, to improve physical condition and increase patient survival. In this study, we recruited and followed up a female NSCLC patient, whose plasma ctDNAs (circulating tumor DNAs), blood cell DNAs, psDNAs (pleural effusion supernatant DNAs), and ppDNAs (pleural effusion pellet DNAs), were collected and analyzed over periodic time points by methods of next generation sequencing (NGS), droplet digital PCR (ddPCR), and Amplification Refractory Mutation System (ARMS). In addition, pleural effusion pellets were stained by IHC (immunohistochemistry). The investigation results showed that EGFR L858R mutation was recognized by methods of NGS, ddPCR, and ARMS, while EGFR T790M mutation was only identified by methods of NGS and ddPCR but not ARMS, indicating that ARMS as an auxiliary clinical diagnostic method, is less sensitive and less reliable than NGS and ddPCR. In summary, the non-invasive and sensitive way of collecting ctDNAs for NGS and/or ddPCR screenings offers patients new diagnosis and therapeutic options.

## Introduction

Lung cancer is a malignant tumor which has the highest incidence and mortality rate among all cancer types worldwide. More than 80% lung cancer cases are non-small cell lung cancer (NSCLC), which can be subdivided into three histological categories: adenocarcinoma (accounts for 40%), squamous cell carcinoma and large cell carcinoma (1). Although continuous emerging of novel diagnostic tools and therapeutic strategies have improved lung cancer treatments, only 18% of lung cancer patients could survive beyond 5-years (2). The main reasons include lack of targeted therapeutic methods and diagnostic methods for early stage, resulting in missing the best chance for treatment.

At present, molecular targeted therapies have been shown great success in NSCLC and other cancer types, the reprehensive and the most prosperous paradigm is by targeting mutation-activated epidermal growth factor receptor (EGFR) in NSCLC patients (3). EGFR mutations have been found in more than 16% of NSCLC patients in western countries, and up to 40% of East Asian NSCLC patients (4, 5). It is reported that more than 80% EGFR mutations in NSCLC are deletions in exon 19 and a point mutation (L858R) in exon 21, which induces the constitutive activation of EGFR in the EGFR mutant cancer cells (6, 7). The therapeutic methods targeting mutation-induced EGFR activation have exhibited great success in clinics for lung cancer patients harboring EGFR mutations, therefore, raising the opportunities to guide the targeted therapies for NSCLC patients who have the EGFR mutations.

Currently, tissue biopsy is the gold standard for assessing tumor molecule changes. However, due to most of NSCLC patients were diagnosed at later stages, it made surgery or biopsy difficult and dangerous, it is reported that about 17% of the cases had accompanying complications of transthoracic biopsy (8). In addition, tumor heterogeneity has been recurrently reported; one or multiple biopsies may not state all the molecular changes of tumor patients due to tumor heterogeneity. Therefore, novel non-invasive and comprehensive investigation and examination methods are urgently need.

Liquid biopsy, including blood, urine, saliva, etc., can be simply collected in a non-invasive or minimally invasive way. As a result, it has attracted researchers and scientists to use liquid biopsy as the newer diagnostic samples. Circulating tumor DNAs (ctDNAs) in peripheral blood circulation are released from tumor cells and contain the genome information of the tumor, have been recommended and investigated as the star biomarkers of liquid biopsy in recent years (9, 10). ctDNAs based diagnostic methods have multiple advantages over other biomaterials. First, the ctDNAs' sampling is non-invasive, could be easily collected by blood draw. Second, ctDNAs contains a pool of tumor genome DNAs of different tumor clones or tumors from different sites, thus ctDNAs investigation could shed lights on all the tumor genomic changes from a patient and resolve the problem of tumor heterogeneity. Third, the measurement of ctDNAs could achieve real-time monitoring of the tumor progression on the molecular level and guide the clinical treatment in time. Fourth, ctDNAs could be investigated in a high-through-put manner, tens to thousands of genomic loci could be analyzed in one examination. Hence, blood ctDNAs detection offers the new opportunity to aid diagnosing, monitoring tumor progression, and guiding the clinical patient treatment, by a non-invasive, real-time, and high-throughput way (11, 12).

In current study, we recruited and followed up a NSCLC patient, and examined her plasma ctDNAs, blood cell DNAs, psDNAs and ppDNAs, using methods of NGS, ddPCR and ARMS. The results showed excellent agreement on the EGFR L858R mutation. However, the EGFR T790M mutation was discovered only by NGS and ddPCR, but missed by ARMS. The discrepancy of sensitivity on the EGFR T790M mutation would disturb the clinical judgment of NSCLC treatment since T790M mutation induces the resistance of the widely used targeting drug gefitinib. As a result, NGS and ddPCR analysis of plasma ctDNAs showed pronounced advantages on sensitivities over ARMS.

## Method

### Patient and ethics

**Figure 1:**
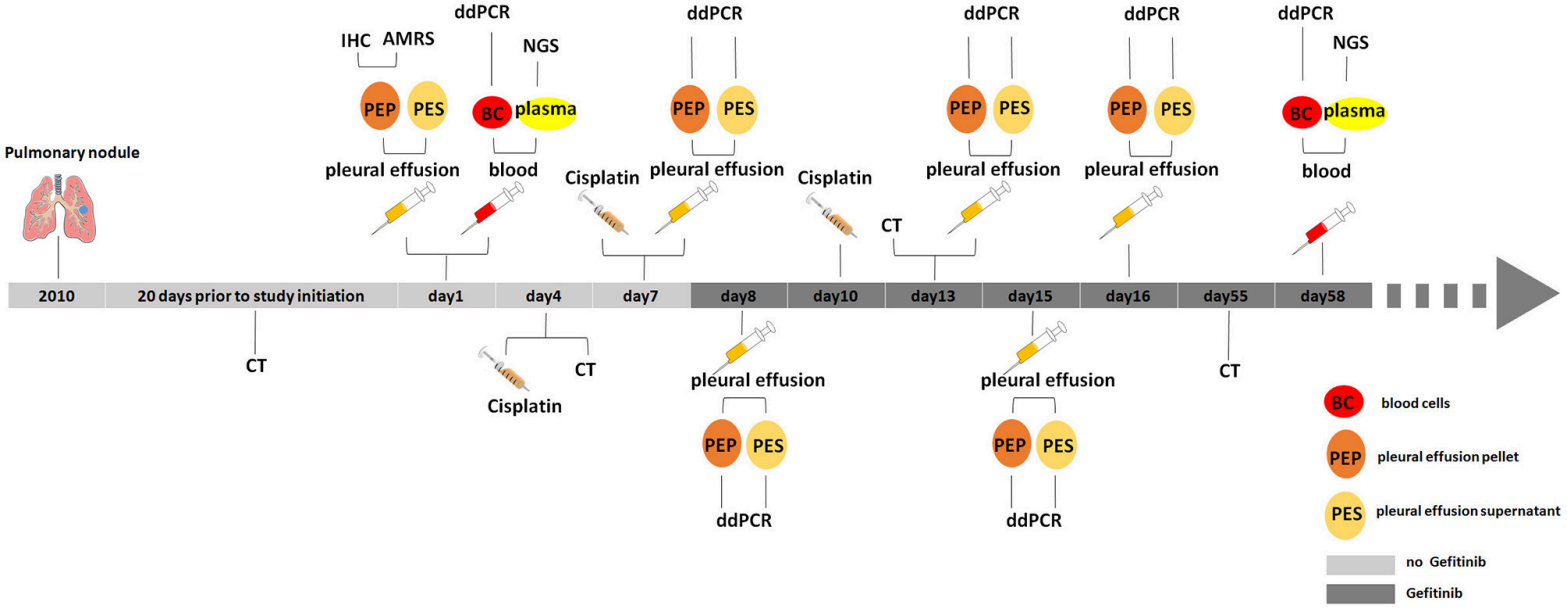
Medical history and treatment for the NSCLC patient. CT, computed tomography; IHC, immunohistochemistry; AMRS, amplification refractory mutation system; ddPCR, droplet digital PCR; NGS, next generation sequencing.

The NSCLC patient (lung adenocarcinoma) is a 75-year-old woman diagnosed and enrolled in this study in the First Affiliated Hospital of Henan University in 2016. This patient has a pulmonary nodule found in her left upper lung in 2010 with no diagnosis of tumor. The mass of pulmonary nodules has obviously increased in 2015-2016 and diagnosed as “upper left pulmonary nodules and left pleural effusion” 20 days before the study started (Figure 1). She has never been given tumor treatment or chemotherapy before the start time of this study. Patient informed consent was acquired and the institutional review board approval was obtained before the start of this study.

### Sample collection and processing

Blood was drawn and collected into K2-EDTA tubes, and then was centrifuged for 10 min at 3,000 g to separate the supernatant plasma from the pellet blood cells. Pleural effusion was also collected in periodic time points as showed in Figure 1, and then centrifuged for 10 min at 3,000 g to separate the pleural effusion supernatant from pleural effusion pellet.

**Figure 2:**
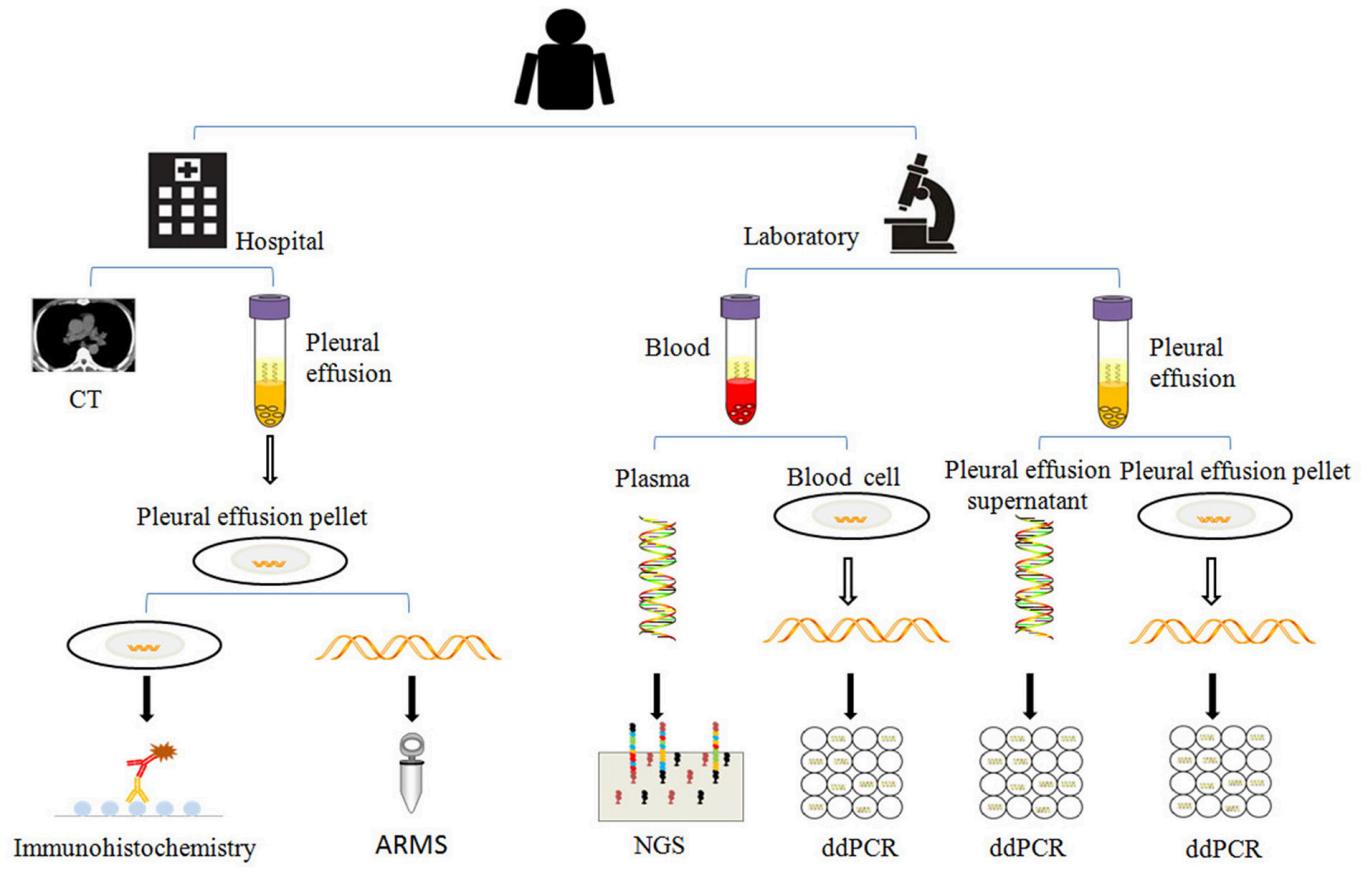
The analysis methods used in this study. When the patient was enrolled, the patient was examined by chest CT (computed tomography), the pleural effusions and blood were collected in regular time points as described in Figure 1, and separated for extraction of psDNAs, ppDNAs, ctDNAs, and gDNAs for ARMS, ddPCR and NGS analysis.

Separated plasma, blood cells, pleural effusion supernatant, and pleural effusion pellet were subject to ctDNAs, gDNAs (blood cell genomic DNAs), psDNAs (pleural effusion supernatant DNAs), and ppDNAs (pleural effusion pellet DNAs) extraction. ctDNAs was extracted with Magnetic Serum/Plasma Circulating DNA Kit (TIANGEN®), gDNAs, and ppDNAs were isolated using SDS-alkaline lysis method and dissolved into 50 μl Tris-EDTA buffer (TE). These samples were stored at −80°C for later detection (Figure 2).

### NGS, ddPCR, and ARMS analysis

ctDNAs were analyzed by Ion Torrent PGM Systems (Life Technologies, USA). The targeted gene panel was designed with the Ion AmpliSeq Designer tool. The library of ctDNAs was constructed using Ion AmpliSeq™ Library Kits 2.0 (Life Technologies, USA) according to manufacturer's instructions. Next, ctDNAs library was clonally amplified by Ion One Touch™ system (Life Technologies, USA). Ion-spherical particles were automatically enriched by Ion One Touch™ ES^+^. Massively parallel sequencing was run on a Personal Genome Machine™ Sequencer (Life Technologies, USA) by the Ion PGM™ Sequencing 200 Kit v2 according to manufacturer's instructions. The sequencing data was analyzed by the Ion Torrent platform-specific pipeline software Torrent Suite v1.4 (Life Technologies, USA). Human GRCh37/hg19 was used for genome reference sequence. The Coverage Analysis plug in was used to assess coverage. Variant Caller tvc-5.0.3 was applied to call variants. Sequence data was visualized by the Integrative Genomics Viewer.

Allele refractory mutation system (ARMS)-based quantitative PCR (qPCR) was performed in clinical laboratory of the First Affiliated Hospital of Henan University (Supplementary Table S1). When NGS and ARMS analysis were finished, EGFR 790th site was found to have different mutation status between NGS and ARMS test. Thus, the 858th and 790th sites of EGFR gene were re-analyzed by the QX200™ Droplet Digital™ PCR System (Bio-Rad Laboratories, USA) in accordance with the manufacturer's instructions, the EGFR 858th/790th site mutation frequency of each sample was reported by calculating the ratio of the positive droplets over the total droplets combined with Poisson distribution.

## Results

### High sensitivity of NGS analysis of ctDNAs over ARMS

In this study, one NSCLC female patient was enrolled, and her pleural effusion and blood samples were collected at the first in-patient day. After centrifuging pleural effusion, the pleural effusion pellet was sent to clinical laboratory and subject to immunohistochemical staining against EGFR. IHC result showed that EGFR is positively stained in pleural effusion pellet. In the meanwhile, blood sample was separated into blood cells and plasma, plasma was used to extract the ctDNAs for NGS, the gDNAs of blood cells were kept in freezer to be used for further test and validation (Figure 1).

**Figure 3:**
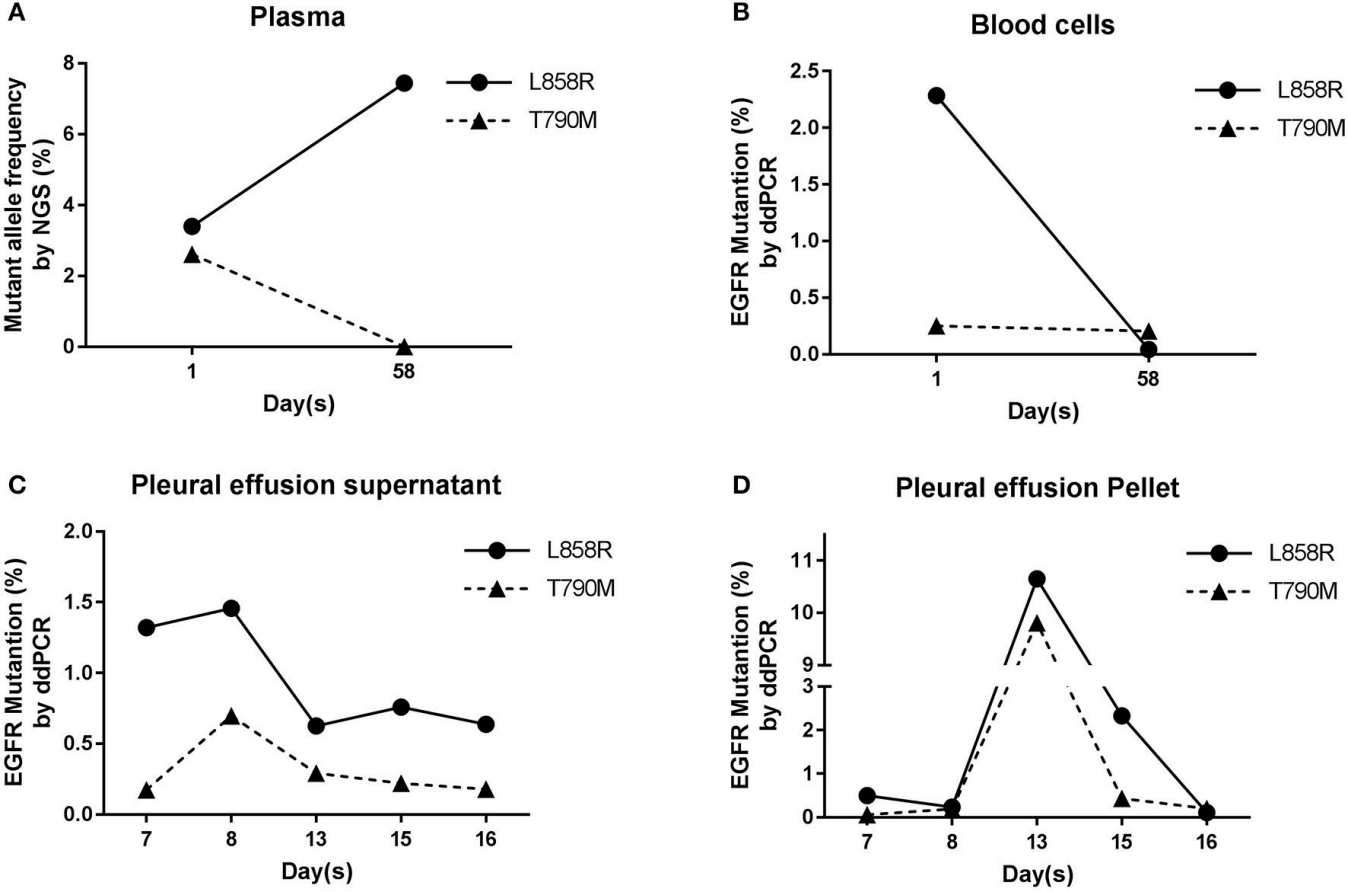
EGFR analysis in blood and pleural effusion samples. **(A)** Results of NGS ctDNAs analysis before and after treatment. **(B)** Analysis of gDNAs by ddPCR. **(C,D)** Dynamic mutation frequency of EGFR in psDNAs **(C)** and ppDNAs **(D)** by ddPCR after the patient had been started to be treated by cisplatin and gefitinib.

Because of the positive EGFR IHC staining result, subsequently EGFR frequent mutation sites, included 19-Del, L858R, T790M, 20-Ins, G719X, S768I, L861Q, were tested by ARMS on ppDNAs. ARMS result showed that this NSCLC patient has EGFR L858R mutation and is wild type in other tested genic sites (Supplementary Table S1). Meanwhile, targeted NGS analysis on plasma ctDNAs was performed (Supplementary Table S2). NGS result of ctDNAs showed that this patient carries not only EGFR L858R mutation (3.4%) but also EGFR T790M mutation (2.61%, Figure 3A). The analysis of ppDNAs by ARMS and ctDNAs by NGS were demonstrated to have distinct mutation examination results on 790th site of EGFR gene.

To further validate and evaluate the results from ARMS and NGS, ddPCR was performed to analyze the 858th and 790th sites of EGFR gene using gDNAs from blood cells, and ddPCR results showed that 2.28 and 0.29% of gDNAs have EGFR L858R and T790M mutations (Figure 3B), respectively, confirming the T790M mutation on EGFR from NGS and indicating the higher sensitivity of NGS analysis than ARMS.

### Real-timely monitoring the tumor progress by NGS and ddPCR

Based on primary diagnosis and clinical test results from IHC and ARMS, the patient was treated by chest injection of Cisplatin at 4th, 7th, and 10th day and given gefitinib from 8th day (Figure 1).

To monitor the tumor progression and effect of patient treatment, we collected pleural effusion samples at 7th, 8th, 13th, 15th, and 16th day and separated pleural effusion supernatant from pleural effusion pellet, and extracted psDNAs (pleural effusion supernatant DNAs) and ppDNAs (pleural effusion pellet DNAs) for ddPCR analysis (Figures 3C,D) on EGFR 858th and 790th sites. The ddPCR results showed that the frequency of both L858R and T790M mutations decreased at 13th day and were kept the comparable levels until 16th day in the pleural effusion supernatant (Figure 3C), in contrast, both L858R and T790M levels increased at 13th day and promptly dropped at 15th day in pleural effusion pellet (Figure 3D).

At the 58th day from enrollment, the blood sample of this NSCLC patient was collected for a second time, and was separated into plasma and blood cells for ctDNAs and gDNAs extraction intended for NGS and ddPCR analysis (Figures 2,3A,B). NGS analysis showed that EGFR L858R and T790M mutation rates were 7.44 and 0%, while ddPCR results presented 0.04 and 0.2% rates for EGFR L858R and T790M mutations.

## Discussion

Tissue biopsy is the gold standard for diagnosis, but is invasive for the patient and may induce the high risk of complications, such as bleeding, pneumothorax, respiratory depression, cardiopulmonary arrest, arrhythmia, and tracheal paralysis (13, 14). In addition, tissue biopsy can only show information in the tissue section that was collected and is likely missing information from other region of the tumor. Therefore, comprehensive and non-invasive diagnosis methods are urgently needed.

In recent couples of years, plasma ctDNAs NGS sequencing has been reported to be highly sensitive, comprehensive, and non-invasive to assist the diagnosis and prognosis of cancers, and to assess tumor progression and tumor load during treatment and real-timely monitor the effect of treatment (15–17). Abbosh et al. reported that ctDNAs sequencing could detect the molecular events of NSCLC relapse 70 days ahead the CT scanning, and could identify the molecular events of different clone/subclones of primary tumor and its metastasis (17). As a result, ctDNAs sequencing had shown great advantages over CT scanning. However, until to now, there is no report to compare the sensitivities of distinct molecular techniques between NGS, ddPCR, and ARMS.

In our study, the pleural effusion pellet of NSCLC patient was positive for EGFR by IHC staining, and the patient was examined to have EGFR L858R mutation and wild-type 790th site by ARMS in clinical laboratory, meanwhile her plasma ctDNAs collected at the same day and were analyzed by NGS. NGS results showed that this patient carries both EGFR L858R and T790M mutations. The discrepancy of test results on EGFR T790M mutation is clinical important and critical for the NSCLC patient treatment, this is because NSCLC patient with EGFR T790M mutation was reported to be resistant to gefitinib (18, 19). As a result, we performed the additional investigation of EGFR gene on 858th and 790th sites by ddPCR, a low-throughput but very sensitive technique. The ddPCR results validated the finding from NGS that the NSCLC patient carries both EGFR L858R and T790M mutations, indicating the high-throughput and sensitive advantages of NGS analysis of ctDNAs over ARMS analysis of ppDNAs.

In conclusion, ctDNAs sequencing is a promising, sensitive, comprehensive and high-throughput technique beyond the traditional tissue biopsy, it offers the opportunities to detect tumor-driven gene mutations, monitor tumor progression/evolution, guide tumor diagnosis, and benefit the clinical precision medicine by a non-invasive way.

## Ethics statement

This study was carried out in accordance with the recommendations of Ethical review of human scientific research and experimental animals, Ethics Committee on medicine and research of Henan University Medical School with written informed consent from all subjects. All subjects gave written informed consent in accordance with the Declaration of Helsinki. The protocol was approved by the Ethics Committee on medicine and research of Henan University Medical School.

## Author contributions

XG, YL, and SW contributed to the conception and design of the study. JL, JX, TX, GZ, and YW collected the samples and performed the research. TL and WZ analyzed data. YT and AL performed the statistical analysis. YL and JL wrote the first draft of the manuscript. All authors contributed to manuscript revision, read and approved the submitted version.

### Conflict of interest statement

The authors declare that the research was conducted in the absence of any commercial or financial relationships that could be construed as a potential conflict of interest.
