# Supplementary material for: High Sensitive and Non-invasive ctDNAs Sequencing Facilitate Clinical Diagnosis And Clinical Guidance of Non-small Cell Lung Cancer Patient: A Time Course Study
Source: Front Oncol. 2018 Oct 30;8:491. doi: 10.3389/fonc.2018.00491 (PMC6218404; doi:10.3389/fonc.2018.00491)
Supplement: Supplementary file 1 [file Table_1.DOCX]

**Supplementary table s1**. EGFR mutation status examined by ARMS analysis in clinical laboratory.

| **Gene** | **Exome** | **Mutation sites** | **M/W^1^** | **Response to EGFR-TKI therapy** |
| --- | --- | --- | --- | --- |
| EGFR | Exon 19 | 19-Del | W | Yes |
|  | Exon 21 | L858R | M |  |
|  | Exon 18 | G719X | W |  |
|  | Exon 20 | S768I | W |  |
|  | Exon 21 | L861Q | W |  |
|  | Exon 20 | T790M | W | No |
|  | Exon 20 | 20-Ins | W |  |

**^1^**Abbreviation: M, mutant; W, wild type.

**Supplementary table s2**.Gene panel of targeted sequencing for circulating tumor DNAs (ctDNAs) analysis by NGS.

| ABL1 | AKT1 | ALK | APC | ATM | BRAF |
| --- | --- | --- | --- | --- | --- |
| CDH1 | CSF1R | CTNNB1 | DDR2 | EGFR | ERBB2 |
| ERBB4 | FBXW7 | FGFR1 | FGFR2 | FGFR3 | FLT3 |
| GNAS | HNF1A | HRAS | IDH1 | JAK2 | JAK3 |
| KDR | KIT | K-RAS | MAP2K1 | MET | MLH1 |
| MRL | NOTCH1 | N-RAS | PDGFRA | PIK3CA | PTEN |
| PTPN11 | RB1 | RET | SMAD4 | SMARCB1 | SMO |
| SRC | STK11 | TP53 | TSC1 | VHL | CDA |
| CYP19A1 | CYP1B1 | CYP2C19 | CYP2C8 | CYP2C9 | CYP2D6 |
| DPYD | ERCC1 | ERCC2 | GSTT1 | MDM4 | MDR1 |
| MRP2 | MTHFR | NQO1 | PON1 | SLC22A12 | TPMT |
| TYMS | UGT1A1 | XRCC1 |  |  |  |

Note: The gene panel included frequent tumor mutations and treatment-response predictive sites.
